# Participants’ Perspectives on Health Impact, Barriers and Facilitators to Adherence in a Mediterranean Diet Lifestyle Trial

**DOI:** 10.3390/nu18010063

**Published:** 2025-12-24

**Authors:** Paloma Massó Guijarro, María Durán-Luque, Claudia Rojas-Medina, Naomi Cano-Ibáñez

**Affiliations:** 1 Department of Preventive Medicine and Public Health, Faculty of Medicine, University of Granada, Avda. Dr. Jesús Candel Fábregas 11, 18016 Granada, Spain; pmasso@ugr.es (P.M.G.); claudiarojas@correo.ugr.es (C.R.-M.); ncaiba@ugr.es (N.C.-I.); 2 Instituto de Investigación Biosanitaria de Granada (IBS.GRANADA), 18012 Granada, Spain; 3 Centro de Investigación Biomédica en Red de Epidemiología y Salud Pública (CIBERESP-Spain), 28029 Madrid, Spain

**Keywords:** Mediterranean diet trial, lifestyles, adherence, barriers, facilitators, qualitative study

## Abstract

**Background/Objectives**: Interventions promoting the Mediterranean Diet (MedDiet) and healthy lifestyle behaviours are effective and cost-efficient in preventing non-communicable diseases (NCDs), yet sustaining adherence remains challenging. This study explored perceived health impacts on, barriers to, and facilitators of adherence among older Spanish adults participating in a randomised clinical trial (RCT) based on the MedDiet and lifestyle interventions. **Methods**: A qualitative study was conducted with 17 Spanish participants (aged 60–81 years) with overweight/obesity and metabolic syndrome (MetS). In-depth, semi-structured interviews were audio-recorded, transcribed verbatim, and analysed through inductive thematic analysis with a gender-sensitive approach. **Results:** Participants identified several facilitators, including perceived improvements in vitality, psychological well-being, and physical performance, alongside enhanced nutritional literacy and confidence in orchestrating daily dietary practices. Women emphasised empowerment, autonomy, and the satisfaction of promoting family health. Main barriers included low motivation, disease burden, mobility restrictions, and limited partner support, with women particularly highlighting emotional and relational factors. A continuous, trust-based relationship with the research team acted as a strong external facilitator supporting long-term commitment. **Conclusions**: Perceived health gains, self-efficacy, social engagement, and research team support facilitated adherence, while low motivation, illness burden, and limited partner involvement hindered it. These findings highlight the importance of gender- and context-sensitive strategies to optimise adherence and the long-term effectiveness of Mediterranean lifestyle interventions.

## 1. Introduction

Non-communicable diseases (NCDs), the leading cause of mortality worldwide, represent a major challenge for public health [1]. Among the NCDs, metabolic syndrome (MetS) has a global prevalence of 31.4% [2,3]. The main causes and comorbidities associated with MetS are largely related to modifiable lifestyle factors, including unhealthy dietary habits and physical inactivity [4,5]. Consequently, public health agencies emphasise the need for preventive, cost-effective strategies to reduce the growing burden of NCDs on healthcare systems [6].

Research efforts have increasingly focused on interventions promoting healthy dietary patterns and lifestyle modifications [7,8,9]. Evidence from randomised clinical trials (RCTs) demonstrates that adherence to the Mediterranean Diet (MedDiet) is among the most effective approaches for preventing and managing MetS and its associated risk factors [10]. In most MedDiet RCTs, higher adherence to dietary recommendations has been strongly associated with improved health outcomes [11,12]. However, a key limitation of such trials lies in the high level of participant motivation required to sustain adherence and complete long-term follow-up [13]. Non-adherence rates of up to 50% have been reported, posing a significant barrier to achieving the anticipated health benefits [14].

Enhancing adherence to dietary and lifestyle interventions requires engaging participants to better understand the enablers and obstacles they face in changing established habits [15]. Despite the robust quantitative evidence supporting the benefits of MedDiet interventions, limited attention has been given to the behavioural, psychosocial, and gender-specific factors that influence adherence over time. Understanding these subjective experiences is essential to bridge the gap between efficacy under controlled trial conditions and real-world effectiveness. Identifying perceived barriers and facilitators within the target population allows researchers and practitioners to adapt intervention protocols, personalise support strategies, and improve long-term adherence and outcomes [16,17].

While quantitative studies have primarily examined the clinical outcomes of MedDiet RCTs [18], qualitative research offers valuable insights into the personal and contextual factors shaping adherence behaviours. This methodological approach provides a deeper understanding of how participants interpret and integrate dietary knowledge acquired during intervention programmes to their daily lives. This study explored perceived barriers and facilitators to adherence among older Spanish adults participating in an RCT based on MedDiet and lifestyle interventions.

## 2. Materials and Methods

### 2.1. Study Design

A qualitative content analysis study based on in-depth semi-structured interviews, ancillary to an ongoing 6-year randomised clinical trial (RCT) on the Mediterranean lifestyle, was conducted (Registration Number 89898870; http://www.isrctn.com/ISRCTN89898870, accessed on 6 November 2025). The RCT aimed to evaluate an intensive lifestyle intervention programme promoting traditional MedDiet, physical activity, and behavioural support for cardiovascular disease prevention, compared with usual care and general dietary counselling. The protocol and main results of the trial have been reported elsewhere [5,19]. This qualitative study followed the Standards for Reporting Qualitative Research (SRQR) [20] to ensure methodological rigour and transparency. In addition, the study adhered to the Sex and Gender Equity in Research (SAGER) guidelines to guarantee the systematic integration of a gender perspective throughout the study design, methodology, and data analysis [21,22] (Supplement S1).

### 2.2. Sample Selection

Participants were selected from the larger randomised clinical trial cohort (*n* = 296) at the Granada research site, following a maximum variation purposive strategy to capture a broad range of experiences [23]. The target population of the trial comprised community-dwelling older adults (men aged 55–75 years, and women aged 60–75 years) with overweight or obesity (BMI between 27 and 40 kg/m^2^), free from cardiovascular disease at enrollment, and diagnosed with MetS.

To achieve diversity within the qualitative sample, participants were chosen to represent differences in sociodemographic factors such as age, gender, marital (widowed, married, divorced, and never married) and occupational status (active, retired, and never working), educational level (primary, secondary, and university), number of cohabitants, and place of residence (urban or rural). Additional inclusion considerations were related to participants’ engagement in the trial, including allocation group, degree of weight change during the first six months, improvement in adherence to MedDiet, motivation to remain in the study, and attendance at follow-up visits. The inclusion of participants from both the intervention and control groups was intentional and consistent with the qualitative aim of capturing a wide range of experiences related to perceived health impact and adherence, rather than assessing intervention effects.

Eligible participants were contacted by telephone and invited to take part in the interviews. The recruitment process was carried out by P.M.-G. (PhD, MD, Anthropologist and Medical Specialist in Preventive Medicine, University of Granada), N.C.-I. (PhD, Nurse, Nutritionist and Lecturer, University of Granada), M.D.-L (Nursing phD student, University of Granada) and C.R.-M. (Nursing student, University of Granada).

### 2.3. Data Collection

Data were gathered between March and May 2023 through 17 individual semi-structured interviews. Each session was conducted in person and lasted approximately 30 to 60 min. The research team comprised two female investigators: N.C.-I., P.M.-G., and one research assistant: C.R.-M. All interviews were conducted at the University of Granada’s Faculty of Medicine (Spain), where participants’ visits during the RCT follow-up were held. The interviewers made a presentation introducing the other researchers to each participant, explaining selection reasons, purpose of the interview, and advising that conversation would be audio-recorded for later transcription ensuring confidentiality. All interviews were audio-recorded, occasionally, recording was stopped at the participants’ request when they felt that some comment was particularly private, or that could be politically incorrect. Records yielded a total of 12.5 h of material for analysis.

A semi-structured interview guide (Supplement S2) with open-ended questions was used to explore participants’ health impact, barriers, and facilitators related to adherence to the Mediterranean lifestyle intervention. This approach facilitated the ‘emic’ perspective [24], minimised researcher bias, and encouraged the emergence of new categories. Interviewers stimulated participants to reflect freely on their participation in the clinical trial and on how they had managed to incorporate dietary and lifestyle recommendations into their everyday routines.

To ensure an open and comfortable atmosphere, participants were informed that there were no right or wrong answers and that their views and experiences were highly valued. During the sessions, one researcher led the discussion, another took field notes documenting non-verbal cues and contextual observations, and the assistant transcribed relevant statements in real time. Immediately after each interview, the team held a brief debriefing meeting to review impressions, discuss data quality, and adjust the interview guide when necessary. Saturation principle was applied to achieve maximum discursive heterogeneity and representativeness. Data collection continued until no new topics of interest emerged, and the information started to become redundant [25].

From a reflexivity standpoint, all the researchers were young women, and N.C.-I., P.M.-G. and M.D.-L. had professional and academic training related to patient care, as well as experience in recruiting and monitoring in multiple studies. In addition, many participants were already familiar with the researchers, as all of them—except C.R.-M.—worked in the setting where the RCT visits and group sessions were carried out. These facts were expected to facilitate rapport and to foster open communication during the interviews. An anthropological perspective was provided by P.M.-G., which contributed to avoiding biomedical bias, and to sustaining more in-depth analytical discussions during the interpretation of the interviews, guided by the gender sensitivity shared among the researchers.

### 2.4. Theoretical and Analytical Framework

In this qualitative study, sociocultural dimension [26,27], and gender perspectives [28,29,30,31,32,33] were applied across the study design, methodology and analysis for addressing processes influencing participants’ lifestyles and adherence to study recommendations. The conceptual framework of thematic analysis was based on the updated MedDiet pyramid, which offers a more holistic view beyond nutritional recommendations, and emphasised social environment, relationships, and physical activity within a Mediterranean lifestyle [34].

### 2.5. Data Analysis

The audio records of 17 interviews were transcribed within two days by P.M.-G. and C.R.-M. A thematic analysis was performed collaboratively by the research team (P.M-G., N.C.-I., and C.R.-M.) [35]. Inductive orientation was used to identify themes and categories related to the research topics on participants’ experiences shaping their adherence to the RCT intervention protocol. Data analysis started simultaneously with data collection, using quotes from fieldnotes and researchers’ insights after each interview with a debrief of main topics, comments, and impressions during the conversations. Audio-records of interviews were transcribed within two days using oTranscribe software (https://otranscribe.com/). To ensure truthfulness and data accuracy, transcriptions were carefully checked against the original recording and field notes taken during interviews.

Constant comparison method was used to code collected data, to segment by themes, and to group information into relational category trees [36], as well as to triangulate data analysis among researchers [37]. After an in-depth reading of each transcription, researchers identified categories and subcategories, which were grouped into main themes according to topic guide and key points in the fieldnotes. All themes, categories, subcategories, and illustrative verbatims were shared among researchers to add, modify, and regroup them, and to include or relocate quotes after each transcription analysis. Disagreements were solved though discussion among these three researchers to choose the categories that best suited the purpose and framework of the study. The emergence of each theme and category was interpreted according to sociodemographic characteristics of participants (i.e., gender, age, civil status, occupation, etc.) to observe differences in their health perceptions and facilitators or barriers to adherence.

### 2.6. Ethical Statement

The study protocol complied with the principles of the Declaration of Helsinki and was approved by the Provincial Ethics Committee of Granada, Spain (Registration number: S1045, 0936_N-22, approval date: 28 April 2023). All participants provided written informed consent. Data protection was ensured in accordance with Organic Law 3/2018 on Personal Data Protection and Regulation (EU) 2016/679 of the European Parliament and of the Council.

## 3. Results

### 3.1. Sociodemographic Characteristics of Participants

**Figure 1:**
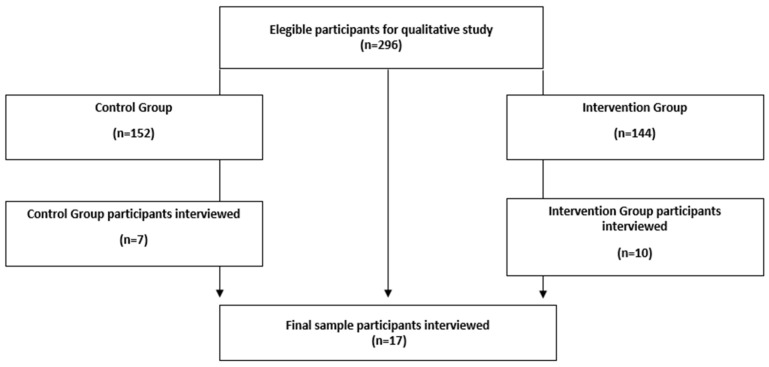
Flow diagram of participant recruitment and inclusion in the qualitative study (final sample *n* = 17).

Among 296 subjects contacted, 17 agreed to participate (10 men and 7 women) (Figure 1). The mean age was 67.4 ± 5.7 years (range 60–81). Most participants were married (*n* = 15, 88.2%), retired (*n* = 16, 94.1%), and resided in urban areas (*n* = 13, 76.5%), sharing their homes with an average of 2.4 cohabitants. Educational attainment was mainly medium (*n* = 6, 35.3%) or low (*n* = 7, 41.2%). Regarding their allocation in the clinical trial, 10 participants (58.9%) belonged to the intervention group and 7 (41.2%) to the control group. Concerning outcomes achieved during participation in the randomised trial, 6 participants (35.3%) reported a weight reduction greater than 5%, while two-thirds (64.7%) showed an improvement in MedDiet adherence after six months of follow-up. Most participants (*n* = 13, 76.5%) attended at least 75% of scheduled follow-up visits. Individuals with higher visit attendance generally achieved greater dietary adherence and weight loss, particularly those assigned to the intervention arm (Table 1).

### 3.2. Thematic Analysis of Interviews

Main themes identified were: (1) experiences of enrollment in MedDiet lifestyle trial study; (2) perceived health impact; (3) Facilitators to adherence with the MedDiet and lifestyle recommendations; (4) Barriers to adherence with the MedDiet and lifestyle recommendations; and (5) opinions and suggestions for improving the intervention.

## 4. Experiences of Enrollment in a MedDiet Lifestyle Trial Study

### 4.1. Motivations for Participation

Participants expressed multiple reasons for joining the study, mainly related to health improvement and interest in learning healthier eating habits (Table S1). A recurring motivation was the desire to increase nutritional knowledge: “Being taught to eat well, because we don’t know how to eat well.” (08399, Woman, 60 years). This intention was closely linked to a sense of self-control and personal growth: “…improving one’s conscience in terms of diet, exercise and leading a healthy life.” (08382, Man, 61 year). A sense of social responsibility and commitment to scientific research was also mentioned: “…if this is a valuable study, first for some people who might fry their eggs with butter, so that they learn not to do so.” (08341, Woman, 63 years). Some women expressed a protective, almost maternal attitude toward the research team, as reflected in: “The first point is collaboration with young people,…so that girls can get scholarships and…have a future.” (08341, Woman, 63 years). Expectations of receiving advice for age-related health concerns were also raised: “We need help…to cope with…all the problems that arise at our age and we…don’t know how to solve them.” (08181, Woman, 70 years). For some men, participation also carried cultural meaning and pride in promoting Mediterranean traditions: “…if we’re going to promote olive oil and the Mediterranean diet, then let’s export the way of life as well.” (08382 Man, 61 years). However, not all participants were initially aware of the study’s scope or aims, as noted by one woman: “I had no idea what it was.” (08093, Woman, 67 years). In a few cases, the long duration of the programme discouraged participation, although satisfaction increased over time (Table S1).

### 4.2. Challenges and Personal Goals

At enrollment, many participants reported having no clear personal objectives, often delegating responsibility for outcomes to the research team (Table S1). Still, some viewed participation as a challenge in discipline and perseverance: “Personally, to carry it out…I’m working on it.” (08200, Woman, 67 years). For others, particularly women, the main goal was maintaining health and autonomy to avoid depending on relatives: “I just want to be healthy and be able to…take care of myself…I don’t want to burden my daughters.” (08381, Woman, 76 years). A few participants expressed frustration after previous unsuccessful weight-loss attempts: “I saw this as a dream…because I was about twenty-something kilos overweight.” (08200, Woman, 67 years). Conversely, others acknowledged abandoning their initial weight-loss objectives during follow-up: “At first, I set myself some goals, but then I didn’t set myself anymore.” (08212, Man, 70 years).

## 5. Perceived Health Impact

### 5.1. High Quality of Life

Many women described their well-being holistically, emphasising autonomy, social activity, and self-fulfilment: “Psychologically, I also feel good, fulfilled with what I am doing with my grandchildren, with the activity. I do not feel like a useless woman…, I help as much as I can.” (08200, Woman, 67 years) (Table 2). Participants frequently attributed improved health and vitality to their involvement in the study: “Thanks to [study], I am alive.” (08032, Man, 81 years); “Coming here has been the best thing that has ever happened to me.” (08093, Woman, 67 years). They also emphasised enhanced self-esteem and autonomy: “The self-esteem of being myself, of preparing my own food…Teaching me to take care of myself…” (08093, Woman, 67 years). Several women valued improvements in physical appearance and self-image: “I feel young, I feel better, lighter, I am not flabby, I can wear clothes that I like, and I feel good.” (08200, Woman, 67 years). Some reported notable physical recovery: “I was on a waiting list for a prosthesis, and I did not have surgery because…I lost weight.” (08032, Man, 81 years). Reduced medication use and closer medical monitoring contributed to their sense of security: “The healthy diet…has balanced my blood pressure and weight very well, and I don’t take pills for cholesterol.” (08200, Woman, 67 years).

### 5.2. Moderate Quality of Life

Participants with stable chronic conditions aimed to preserve health and functionality: “I feel fine, I have my ailments, but as long as you keep moving with light physical activity and keep yourself under control, that’s it.” (08382, Man, 61 years). Others prioritised independence over aesthetics: “…I have perhaps paid more attention to ensuring that…I do not become useless in being able to walk…[rather] than what figure I had.” (08381, Woman, 76 years) (Table 2).

### 5.3. Low Quality of Life

A few participants described worsening health and well-being, often associated with disease progression or adverse family circumstances. New cardiovascular diagnoses generated uncertainty and discouragement: “But this year life changed. I have a cardio issue … you start to see yourself as worse off than you really are.” (08212, Man, 70 years). Feelings of loss and depression were also present: “And now I feel useless, I feel depressed because [blindness] has come on almost suddenly.” (08032, Man, 81 years). Social and relational problems further affected their well-being: “[I] was no longer any good for anything, married life [he meant sexual impotence]. I couldn’t do anything.” (08032, Man, 81 years). Several participants also reported sleep problems, family stress, or social isolation affecting adherence and mood (Table 2).

### 5.4. Health Impact on Relatives and Social Networks

Participants often described themselves as agents of change, transferring acquired health habits to family members and neighbours: “I have tried… to pass on the good that I have received.” (08200, Woman, 67 years) and even reported vicarious benefits: “My daughter has taken advantage of that, my son too, and my neighbors.” (08200, Woman 67 years) (Table 2).

## 6. Facilitators to Adherence with the Mediterranean Diet and Lifestyle Recommendations

### 6.1. Nutritional Learning and Behavioural Reinforcement

Participants emphasised that repeated nutritional guidance and individualised feedback strengthened their dietary awareness and confidence to maintain healthy habits (Table 3). Continuous reinforcement through group sessions and follow-up visits facilitated internalisation of key messages: “…you get used to it … because you keep telling us things very repeatedly.” (08212, Man, 70 years). Perceived health improvements, such as weight loss, better mobility, and disease control, acted as strong motivators for sustained adherence: “I did everything exactly as instructed [and]…I noticed the improvement.” (08032, Man, 81 years).

### 6.2. Supportive Relationship with the Research Team

A close, trust-based relationship with the research team emerged as a key facilitator of adherence. Participants valued the team’s empathy, availability, and personalised attention, which fostered commitment and a sense of belonging: “Everything was attentive, delicate, personal encounters…they helped me so much.” (08200, Woman, 67 years). Group sessions also contributed to emotional well-being and empowerment: “They have taught me to go out and … communicate, move forward…and learn self-esteem, enthusiasm.” (08093, Woman, 67 years). Some participants expressed their intention to maintain healthy habits beyond the study: “Everything you have taught me; I will continue to do. I am not going to say, ‘I have finished studying and I am stopping.’” (08347, Man, 69 years) (Table 3).

### 6.3. Sense of Responsibility and Social Meaning

Engagement was often reinforced by a sense of moral duty and gratitude for being part of a meaningful scientific initiative: “If I’m here, it’s to participate seriously…I’m doing serious work [in such relevant study].” (08382, Man, 61 years). The perception of contributing to broader social benefit enhanced participants’ motivation to adhere to and complete the programme. As well, meals and daily activities were often ritualised around their closer social network, as partners, friends, or family members, that reinforced adherence to healthy lifestyles: “I usually get together with a group of…friends…” (08027 Woman, 67 years) (Table 3).

## 7. Barriers to Adherence with the Mediterranean Diet and Lifestyle Recommendations

### 7.1. Loss of Motivation and Difficulty Sustaining Discipline

Several participants acknowledged inconsistency in maintaining healthy habits. Fatigue, low willpower, and entrenched routines were frequently cited as obstacles: “…my fault is that I don’t follow a strict diet like … my brother [also a participant] did.” (08212, Man, 70 years). The challenge of sustaining dietary effort over time was apparent: “[Losing 4 kg] is very difficult…because that would require an additional effort …, the habits I have incorporated are not enough.” (08241, Man, 60 years) (Table 4).

### 7.2. Cultural and Emotional Resistance to Change Eating Habits

Men described difficulty abandoning lifelong eating customs and “food addictions”: “It takes a lot of effort, because we’re already addicted … You can’t cut things out immediately … At my age, it’s very difficult to break old habits.” (08212, Man, 70 years). The “carpe diem” mentality associated with aging often weakened motivation: “There comes a time when I’m not going to stop doing what I like.” (08212, Man, 70 years) (Table 4).

### 7.3. Physical Limitations

Health problems often limited engagement in physical activity and healthy routines (Table 4). Chronic conditions, joint pain, frailty, and apprehension after receiving a diagnosis of severe disease restricted mobility: “I … have a total knee prosthesis, … I can’t walk as fast as I used to walk.” (08212, Man, 70 years). Low public availability of senior-oriented exercise programmes was also mentioned as constraints.

### 7.4. Gendered Challenges and Relational Barriers

For women, caregiving obligations were a recurrent barrier: “I signed up to do sport…, and I had to cancel [because]… I must look after my grandson.” (08093, Woman, 66 years). Many reported frustration with their partners’ non-compliance, which undermined household adherence: “I call it a problem, gluttony…In fact, he’s put on 20 kilos lately … Now I let it go because it’s his freedom and I’m not going to be like a police officer.” (08200, Woman, 67 years). Lack of partner involvement in sharing physical activity was also reported: “I say [to him]: ‘Let’s go out, let’s join a gym…’ [He answered]: ‘No, because you walk too slowly.” (08181 Woman, 70 years). For some widows, adherence was sustained largely through the research team’s support, suggesting external rather than intrinsic motivation: “Once this research is over, I may not be able to lose weight on my own. (…) I’ll have to prepare myself mentally for … and resign myself to them calling me from time to time.” (08027, Woman, 67 years) (Table 4).

## 8. Participants’ Opinions and Suggestions for Improving the Intervention

### 8.1. Overall Satisfaction and Trust in the Research Design

Most participants expressed a high level of satisfaction with the study, describing the experience as positive and well-organized. No substantial changes were suggested, and many perceived the study as exemplary in its planning and implementation: “I can’t find any faults. For me, everything I’ve seen is very, very, very positive…that it’s a real gift, how it’s been planned … very well studied and with experience.” (08200, Woman, 67 years). A generalized attitude of conformity and deference toward researchers emerged, reflecting participants’ confidence in scientific authority and self-perceived lack of expertise to critique methodological aspects: “No, I don’t consider myself competent for that.” (08241, Man, 60 years). The delegation of responsibility to the research team was recurrent: “You will know why it is planned and why certain things are done and so on.” (08200, Woman, 67 years). Conversely, participants often attributed potential shortcomings to themselves rather than to the study: “The only flaw here is that we have it ourselves, that we are very greedy.” (08212, Man, 70 years) (Table S2).

### 8.2. Unmet Expectations and Perceived Gaps in the Intervention

Despite the overall satisfaction, several participants expressed unmet needs, primarily regarding psychological and communicative dimensions of the intervention. Some expected broader support to address aging-related issues: “I thought we were going to get some help with…age-related problems…, a psychologist… But…this [intervention] has only been about nutrition.” (08181, Woman, 70 years). Others found the technical language used in group sessions difficult to follow: “I find it very technical, which might make it difficult for many people to understand.” (08212, Man, 70 years). Logistical difficulties were also cited, particularly balancing study attendance with family caregiving responsibilities: “What has been difficult for me … has been … when … we had to look after our grandchildren and couldn’t attend some meetings … it made me a little angry (…) But it was something personal.” (08200, Woman, 67 years) (Table S2).

A few participants mentioned limited social interaction among participants: “We see each other at meetings, but that’s all.” (08399, Woman, 60 years). Others requested clearer communication of test results and study findings: “I would prefer that, well, since we’ve been there undergoing so many tests, that they at least tell us something about results … if something serious comes up.” (08181, Woman, 70 years); “What is the basis of this study?” (08181, Woman, 70 years) (Table S2).

## 9. Discussion

This qualitative study explored older adults’ perceptions of health impacts, barriers, and facilitators to adherence within a long-term MedDiet and lifestyle randomised clinical trial. Semi-structured interviews provided in-depth insights into participants’ experiences after six years of follow-up, allowing for a nuanced interpretation of how behavioural, social, and contextual factors influenced sustained engagement with the intervention. The analysis revealed that adherence was not merely determined by individual willpower or knowledge, but rather by a complex interplay between health perceptions, psychosocial resources, and relational dynamics.

Most participants reported meaningful improvements in health, vitality, and self-esteem, attributing these gains to enhanced adherence to the MedDiet and increased awareness of healthy living. These perceived benefits, mainly highlighted by women, acted as strong motivators for continued engagement, consistent with prior studies linking perceived health improvements to adherence in lifestyle interventions [38,39]. For women, in particular, participation was associated with a sense of empowerment, autonomy, and self-worth, reflecting gendered patterns of health motivation documented in Mediterranean and aging populations [40]. Moreover, the emergence of participants as informal health promoters within their families and communities underscores the social diffusion potential of such interventions [41,42,43]. However, a gradient in perceived health benefits was observed, conditioned by chronic disease burden, physical limitations, and emotional well-being. Participants experiencing illness exacerbation or frailty often reported decreased motivation and a reduced sense of control over their health, which in turn hindered adherence. This aligns with evidence indicating that multimorbidity and psychological distress negatively influence sustained engagement in dietary and physical activity programmes [44].

Adherence facilitators included increased nutritional literacy, internalized behavioural reinforcement, strong social network, and the supportive relationship developed with the research team. Participants frequently cited empathy, individualized attention, and the continuity of professional contact as key enablers of motivation, reflecting the importance of therapeutic alliance and trust-building in long-term behavioural change interventions [45]. The sustained reinforcement of messages during follow-up visits promoted both accountability and a sense of belonging to the study, particularly among women who valued emotional connection as part of their adherence experience. This was consistent with a systematic review on gender differences to engage in clinical trials, which showed that women’s motivation increased when they perceived rapport from research team, and a conductive atmosphere to express themselves and to feel heard [46]. Moreover, the female gender of the research team was specifically valued by women participants in our study, similarly to recent studies that confirm that there were more female participants in cardiovascular disease clinical trials led by female investigators [47,48].

Additionally, participants highlighted the moral significance of contributing to a meaningful scientific endeavour. In turn, meaningful daily activities ritualized around closer social network [49,50] encouraged them to maintain adherence and to achieve weight goals [51]. This sense of collective purpose and social responsibility reinforced their engagement and may represent a culturally embedded dimension of Mediterranean social identity, where participation in research was perceived as a civic and intergenerational contribution.

Despite these positive factors, several barriers to adherence emerged. Fatigue, low motivation, and difficulties sustaining dietary discipline over time were frequent, mirroring findings from other long-term interventions where initial enthusiasm diminishes without ongoing reinforcement [52]. For many men, resistance to changing long-standing eating patterns or adopting restrictive diets reflected cultural attachment to traditional food practices and a “carpe diem” mindset associated with ageing. Women, conversely, emphasised relational and household barriers, particularly the lack of partner support or shared commitment to dietary change, which often undermined adherence efforts within couples. These findings echo previous research showing that spousal dynamics and gender roles significantly shape lifestyle behaviours in older adults with chronic diseases [53,54,55]. Physical limitations due to chronic conditions or injuries, as well as caregiving responsibilities, further constrained both dietary and physical activity adherence. In Mediterranean cultures, where intergenerational caregiving is common, competing family duties, especially among grandmothers, constitute a recognised challenge to health self-management [56].

### Strengths and Limitations

This study provides novel qualitative insights into the long-term adherence to a Mediterranean Diet and lifestyle intervention among older adults with metabolic syndrome. Strengths include the in-depth exploration through semi-structured interviews, the use of a gender-sensitive and socio-contextual analytical approach, and the triangulation among multidisciplinary researchers, which enhanced credibility and rigor. The inclusion of participants with diverse backgrounds enriched the findings and reflected real-world variability in adherence behaviours. Nevertheless, the study’s qualitative nature and small sample limit generalizability. Selection bias toward more motivated participants and the post-pandemic context may also have influenced reported behaviours. Despite these limitations, the findings yield valuable implications for designing more sustainable, person-centred Mediterranean lifestyle interventions.

## 10. Conclusions

This qualitative study elucidated the perceived mechanisms underpinning adherence to a MedDiet-based lifestyle intervention among older adults with MetS. Perceived health improvements, self-efficacy, social engagement, and the relational support of the research team acted as key facilitators, whereas declining motivation, illness burden, and limited partner engagement hindered sustained adherence. These results highlight that adherence extends beyond nutritional compliance, encompassing psychosocial, gendered, and contextual dynamics that shape participants’ capacity for behavioural change.

Future intervention studies should employ tailored, gender-sensitive motivational strategies involving participants’ relatives and social networks to enhance adherence and maximize the impact of lifestyle programmes in aging populations. Moreover, translating these programmes into healthcare settings remains a challenge despite prior implementations in other regions. This process could benefit from training primary care personnel to lead group sessions using a systemic, gender-sensitive approach that actively engages patients and their families, while ensuring job stability to maintain continuity of care and a strong therapeutic alliance. Finally, practical guides and educational materials could be strengthened by including vignettes or scenarios designed from a gender-sensitive perspective to illustrate everyday challenges and opportunities for improving lifestyles.

## Figures and Tables

**Table 1:** Baseline characteristics and adherence-related outcomes of the study participants.

Code	Sex ^1^	Age (Years)	Marital Status	Educational Level ^2^	Occupation	No. Cohabitants	Type Housing	RTC Allocation Group ^3^	Weight Loss (%) ^4^	Visits’ Attendance ^5^	MedDiet Adherence Improvement ^6^
08007	M	65	Married	Medium	Retired	2	Urban	I	6.8	50	+6
08027	F	67	Widowed	Low	Retired	5	Urban	I	2.3	75	+3
08032	M	81	Married	Low	Retired	2	Rural	I	2.7	75	0
08093	F	66	Widowed	Medium	Retired	3	Urban	C	3.3	100	+2
08181	F	70	Married	Medium	Retired	2	Urban	C	1.1	100	−6
08200	F	67	Married	Low	Retired	2	Urban	I	13.5	83	+5
08212	M	70	Married	High	Retired	1	Rural	I	20.7	83	+4
08231	M	62	Married	Medium	Retired	2	Urban	I	12.2	17	0
08241	M	60	Married	High	Active	2	Urban	I	1.1	33	0
08259	M	68	Married	Low	Retired	2	Rural	I	1.3	100	+4
08341	F	63	Married	Low	Retired	4	Rural	C	−1.5	100	−1
08347	M	69	Married	Medium	Retired	3	Urban	C	−7.5	100	0
08351	M	74	Married	Low	Retired	2	Urban	C	0.3	100	+3
08381	F	76	Married	High	Retired	1	Urban	I	1.0	100	+1
08382	M	61	Married	High	Retired	2	Urban	C	−3.0	100	−2
08399	F	60	Married	Low	Retired	2	Urban	C	5.7	100	9
08412	M	67	Married	Medium	Retired	2	Urban	I	3.4	5	+3

^1^ Sex: M = Male; F = Female. ^2^ Educational level: Low (Elementary studies); Medium (Secondary School); High (University studies and higher vocational training). ^3^ RCT allocation group: I = Intervention group; C = Control group. ^4^ Weight loss (%): (initial weight-final weight/initial weight) × 100. ^5^ No. of visits: (no. of visits attended/no. of total visits scheduled in the study) × 100. ^6^ Index of improved MedDiet adherence: adherence 6 months-baseline adherence/baseline adherence.

**Table 2:** Theme: Perceived health impact.

Category	Subcategory	Verbatim Quotes
2.1. High quality of life	Autonomy and active, social, and meaningful life	“Psychologically, I also feel good, fulfilled with what I am doing with my grandchildren, with the activity. I do not feel like a useless woman who is of no use, I help as much as I can and as I can (…) Life has taught me that you learn from mistakes, you learn from failures and that you should never throw in the towel and move forward.” (08200, Woman, 67 years)
	Gratefulness for health enhancement and survival	“So, thanks to this, I am alive, plain and simple. If I hadn’t died already, if I had continued on the path I was on (…) because I ate too much and things that weren’t good for me… thanks to [the study], I am alive, it’s that simple.” (08032, Man, 81 years) “Coming here has been the best thing that has ever happened to me. I am very happy.” (08093, Woman, 67 years) “Well, I have felt very good [in the study] these seven or eight years.” (08347, Man, 69 years)
	Improvement in eating habits, concern for health, self-care, self-esteem	“Well, what I liked most [about the study] is that it teaches you to eat healthier. It makes you realize what you’re used to eating and encourages you to change.” (08351, Man, 74 years) “The self-esteem of being myself, of preparing my own food. (…) Teaching me to take care of myself, to eat, to know how to behave properly and do things better.” (08093, Woman, 67 years) “I think so, it’s good because it teaches you, first, to get out of the house a little bit. (…) It has helped me to think that I must take care of myself.” (08093, Woman, 67 years)
	Better physical appearance and self-image	“I am very happy with my physical condition because I feel young, I feel better, lighter, I am not flabby, I can wear clothes that I like, and I feel good.” (08200, Woman, 67 years) “When I enter [the meeting room] and see so many old people, I say,… I look better than 90%.” (08381, Woman, 76 years)
	Improvement in disease status and reduction in medical prescriptions	“It has balanced my blood pressure and weight very well, and I don’t take pills for cholesterol.” (08200, Woman, 67 years) “I was on a waiting list for a prosthesis, and I did not have surgery because the pain in my knee went away when I lost the weight.” (08032, Man, 81 years)
	Better medical surveillance and confidence in the research team	“They treated me very well, and the things you do here you don’t do at the [doctor’s] insurance.” (08259, Man, 68 years) “I felt more confident (...) I think there is a remedy because I feel confident with you.” (08032, Man, 81 years)
2.2. Moderate quality of life	Maintenance of health status and disease control	“I feel fine, I have my ailments, but as long as you keep moving with light physical activity and keep yourself under control, that’s it.” (08382, Man, 61 years) “All things considered, I don’t think I’m doing too badly, am I? I’m about to have a catheter fitted next week, but anyway.” (08351, Man, 74 years)
	No concern for physical appearance, but rather for autonomy	“I have perhaps paid more attention to ensuring that I do not lose my memory, that I do not become useless in being able to walk, because I do not want to give my daughters that problem.” (08381, Woman, 76 years)
2.3. Low quality of life	Recent severe diseases and depression	“But this year life changed. I have a cardio issue that is going to complicate me and so I have neither the need nor the desire to put effort into these things [healthy habits].” (08212, Man, 70 years) “And now I feel useless, I feel depressed because [blindness] has come on almost suddenly.” (08032, Man, 81 years)
	Problems with relationships and isolation	“[He changed his lifestyle, gave up tobacco, alcohol and soft drinks because he] was no longer any good for anything, married life. I couldn’t do anything.” (08032, Man, 81 years) “We stopped going out many years ago… our friends have disappeared, because some of them have died.” (08181, Woman, 70 years)
2.4. Health impact on relatives and social networks	Health promotion agency and vicarious health benefits	“I think so, because since I cook [healthy] food for myself, we all eat it.” (08027, Woman, 67 years) “My son used to smoke a lot, but now he doesn’t smoke as much; he’s quitting. (…) My daughter has taken advantage of that, my son too, and my neighbours.” (08200, Woman, 67 years)

**Table 3:** Theme: Facilitators to adherence with the Mediterranean Diet and lifestyle recommendations.

Category	Subcategory	Participants’ Verbatim Quotes
3.1. Nutritional learning and behavioural reinforcement	Awareness, responsibility, commitment, and privilege to be involved in relevant research	“I have been responsible because I consider what I am receiving here at [MedDiet trial] to be beneficial; the diet is very healthy.” (08200, Woman, 67 years) “When the study ends, I will feel satisfied that I have contributed, and that’s it, quite simply. When I started, I knew I would finish, unless I died in the pandemic, you know? I mean, when I commit to something, I carry on to the end (…) I have never thought about giving up or abandoning it. No, no.” (08341, Woman, 63 years) “No, because difficult things always take more effort, but you try to correct yourself, and I’ve liked this from day one.” (08093, Woman, 67 years) “Being here is (…) to give it coherence. If I’m here, it’s to participate seriously, and that commitment has come to me as a result. (…) There will be people who come here, I don’t know, to entertain themselves, but that doesn’t make much sense either, [coming] once every six months isn’t that much fun. But that doesn’t mean I’m not aware that I’m doing serious work, I know [this study] is multinational.” (08382, Man, 61 years)
	Health improvement feedback and clear and adapted recommendations	“I didn’t find anything you explained to me difficult, because I did everything exactly as instructed. Well, I didn’t weigh things, but I did calculate. (…) I didn’t find it difficult at all. I thought everything was fine, because when I did it, I noticed the improvement. I didn’t find anything difficult.” (08032, Man, 81 years)
3.2. Supportive relationship with research team	Appreciation for research team support and empathy	“And it turns out that [attending the meetings] was a pleasant surprise, because everything was attentive, delicate, personal encounters, analytical… That was a huge gift for me, all the people [researchers on the team] I met (…) I will always remember them, honestly, because they helped me so much. Now [with the new researchers] too, yes, and the other one, what’s her name? I don’t want to forget her name.” (08200, Woman, 67 years) “They treated me very well.” (08399, Woman, 60 years) “The people I have had the opportunity to spend time with are very nice and have always treated me very well, etc. So, I’m fine.” (08382, Man, 61 years) “I really liked the meetings, the way they talked to us and presented things to us. Apart from that, they are wonderful people.” (08027, Woman, 67 years) “The study has been good, because, well, all the years I’ve been here, you’ve been looking for us.” (08181, Woman, 70 years)
	Emotional support and motivation for self-esteem	“They taught me to go out and have self-esteem, to be enthusiastic, that’s why I call them ‘my girls’ because they helped me to go out and communicate and move forward and learn (…) They called me the girls who gave us the talks and told us how we had to do things. So that gave me self-esteem, taught me how to eat differently (…). (…) this half hour I spend with you means disconnecting from the four walls and that fills me with life. (…) When you call me, I’m happy. [With the team] I am very happy, they have been the best thing for me. I miss you. I have felt supported, and I really liked seeing that there are people who help me with what I need.” (08093, Woman, 67 years) “From the study [I would highlight] (…) not only the diet, [but also] the enthusiasm with which [the researchers on the team] always brought us to the meetings to encourage us, their patience in repeating things, not taking them for granted, their kindness, their psychological insight (…) Everything was very peaceful, with well-explained slides, repetition, and they gave us the opportunity to ask questions. They were patient with some cases that were unclear and insisted on one meeting, then another, and there was always the same spirit of tolerance, of explaining it again.” (08200, Woman, 67 years)
	Adaptation of communication and educational approach	“You give the sessions in a very generalised way, from a lower cultural level, a higher level and a not very high level, because you understand that our culture is not about teachers, but rather about housewives, a rather low-level culture, at least the people I have seen. So, it is very well adapted.” (08200, Woman, 67 years)
	Wish to maintain healthy habits after the end of study	“Everything you have taught me; I will continue to do. I am not going to say, ‘I have finished studying and I am stopping.’ What I have learned, I will continue to do.” (08347, Man, 69 years) “It’s a shame it’s coming to an end, as you’ll have to continue following the same rules you’ve tried to learn.” (08351, Man, 74 years)
3.3. Sense of responsibility and social meaning	Moral engagement and reflection on self-control	“Whether the programme works [for participants] is a matter of commitment.” (08200, Woman, 67 years) “I have tried to apply what I have learned and pass it on to others, because the important thing is to share what one receives.” (08032, Man, 81 years)
	Ritualised meals and daily activities around closer social network	‘I often go for walks with her [my wife]’ (08241, Man, 60 years, married); ‘I usually get together with a group of … friends…’ (08027, Woman, 67 years, widowed, retired); ‘I have a sister who was widowed … I go every morning … to walk with her’. (08381, Woman, 76 years, widowed, retired).

**Table 4:** Theme: Barriers to adherence with the Mediterranean Diet and lifestyle recommendations.

Category	Subcategory	Participants’ Verbatim Quotes
4.1. Loss of motivation and difficulty sustaining discipline	Inconsistency and lack of determination	“…my fault is that I don’t follow a strict diet like … my brother [also a participant] did.” (08212, Man, 70 years) “[Losing 4 kg] is very difficult…because that would require an additional effort …, the habits I have incorporated are not enough.” (08241, Man, 60 years) “I don’t eat badly, but of course, there are things that I shouldn’t eat, and I do. (…) If I’m not losing weight, I’m not going to get obsessed either.” (08351, Man, 74 years)
	Fatigue and loss of enthusiasm	“At first, I was more motivated, but now I have less willpower; it’s tiring always being on a diet.” (08181, Woman, 70 years) “I do it but not like in the beginning; there are times when I slack off.” (08027, Woman, 67 years)
4.2. Cultural and emotional resistance to change eating habits	Difficulty breaking lifelong eating habits	“It takes a lot of effort, because we’re already addicted … You can’t cut things out immediately, because it might make things even worse … At my age, 74, it’s very difficult to break old habits.” (08212, Man, 70 years, married) “I have a problem, … I always have a big appetite…I really enjoy food, and I care a lot about it.” (08241, Man, 60 years)
	Emotional attachment and pleasure from food	“Food is one of the few pleasures left in life, and at our age, we shouldn’t deprive ourselves too much.” (08382, Man, 61 years) “There comes a time when I’m not going to stop doing what I like.” (08212, Man, 70 years) “Especially at my age, you say, ‘Let’s see if you haven’t had one [beer] today and tomorrow, you’re dead and you can’t have one either,’ right?” (08231, Man, 62 years)
4.3. Physical limitations	Health-related constraints and frailty	“I … have a total knee prosthesis, … I can’t walk as fast as I used to walk. It is something that weighs heavily on me and that I have not adapted … [since] almost a year.” (08212, Man, 70 years) “I used to have a land, but now I’ve given it up because I’ve been having such a rough time … and I wasn’t feeling well, so I sold it.” (08032, Man, 81 years)
4.4. Gendered challenges and relational barriers.	Caregiving obligations	“This year I signed up to do sport…, and I had to cancel [because]… I must look after my grandson … I must think about going out for longer because my joints and bones are crying out for it.” (08093, Woman, 66 years). “I can’t always attend meetings or go walking, because I’m taking care of my grandchildren.”
	Partners’ non-compliance and lack of involvement	“I call it a problem, gluttony…In fact, he’s put on 20 kilos lately … Now I let it go because it’s his freedom and I’m not going to be like a police officer.” (08200, Woman, 67 years). “He says he hasn’t worked his whole life to retire and now, to give up the few things he enjoys”. (08399 Woman, 60 years) “I say [to him]: ‘Let’s go out, let’s join a gym…’ [He answered]: ‘No, because you walk too slowly.” (08181 Woman, 70 years).
	Dependence on research team’s support	“Once this research is over, I may not be able to lose weight on my own. (…) I’ll have to prepare myself mentally for … and resign myself to them calling me from time to time.” (08027, Woman, 67 years).

## Data Availability

The data presented in this study are available upon reasonable request from the corresponding author. The data are not publicly available due to privacy or ethical restrictions.

## Supplementary Materials

The following supporting information can be downloaded at: https://www.mdpi.com/article/10.3390/nu18010063/s1, Table S1: Theme: Experiences of enrollment in MedDiet lifestyle trial; Table S2: Theme: Participants’ opinions and suggestions for improving the intervention. Supplement S1: Sex and gender equity in research guidelines checklist; Supplement S2: Topic guide of semi-structured interviews to participants in the qualitative study.

## Abbreviations

The following abbreviations are used in this manuscript:

BMI	Body Mass Index
C	Control group
F	Female
I	Intervention group
ISRCTN	International Standard Randomised Controlled Trial Number
M	Male
MedDiet	Mediterranean Diet
MetS	Metabolic Syndrome
NCDs	Non-communicable diseases
No.	Number
RCT	Randomised Clinical Trial
SRQR	Standards for Reporting Qualitative Research

## References

[1] World Health Organization (2025). World Health Statistics 2025: Monitoring Health for the SDGs, Sustainable Development Goals.

[2] Noubiap J.J., Nansseu J.R., Lontchi-Yimagou E., Nkeck J.R., Nyaga U.F., Ngouo A.T., Tounouga D.N., Tianyi F.L., Foka A.J., Ndoadoumgue A.L. (2022). Geographic Distribution of Metabolic Syndrome and Its Components in the General Adult Population: A Meta-Analysis of Global Data from 28 Million Individuals. Diabetes Res. Clin. Pract..

[3] Alberti K.G.M.M., Eckel R.H., Grundy S.M., Zimmet P.Z., Cleeman J.I., Donato K.A., Fruchart J.C., James W.P.T., Loria C.M., Smith S.C. (2009). Harmonizing the Metabolic Syndrome: A Joint Interim Statement of the International Diabetes Federation Task Force on Epidemiology and Prevention; National Heart, Lung, and Blood Institute; American Heart Association; World Heart Federation; International Atherosclerosis Society; And International Association for the Study of Obesity. Circulation.

[4] Hernández Ruiz de Eguilaz M., Batlle M.A., Martínez de Morentin B., San-Cristóbal R., Pérez-Díez S., Navas-Carretero S., Martínez J.A. (2016). Cambios Alimentarios y de Estilo de Vida Como Estrategia En La Prevención Del Síndrome Metabólico y La Diabetes Mellitus Tipo 2: Hitos y Perspectivas. An. Sist. Sanit. Navar..

[5] Fernandez-Lazaro C.I., Toledo E., Buil-Cosiales P., Salas-Salvadó J., Corella D., Fitó M., Martínez J.A., Alonso-Gómez Á.M., Wärnberg J., Vioque J. (2022). Factors Associated with Successful Dietary Changes in an Energy-Reduced Mediterranean Diet Intervention: A Longitudinal Analysis in the PREDIMED-Plus Trial. Eur. J. Nutr..

[6] Nugent R., Bertram M.Y., Jan S., Niessen L.W., Sassi F., Jamison D.T., Pier E.G., Beaglehole R. (2018). Investing in Non-Communicable Disease Prevention and Management to Advance the Sustainable Development Goals. Lancet.

[7] Lichtenstein A.H., Appel L.J., Vadiveloo M., Hu F.B., Kris-Etherton P.M., Rebholz C.M., Sacks F.M., Thorndike A.N., Van Horn L., Wylie-Rosett J. (2021). 2021 Dietary Guidance to Improve Cardiovascular Health: A Scientific Statement from the American Heart Association. Circulation.

[8] Godos J., Zappalà G., Bernardini S., Giambini I., Bes-Rastrollo M., Martinez-Gonzalez M. (2017). Adherence to the Mediterranean Diet Is Inversely Associated with Metabolic Syndrome Occurrence: A Meta-Analysis of Observational Studies. Int. J. Food Sci. Nutr..

[9] Virani S.S., Alonso A., Aparicio H.J., Benjamin E.J., Bittencourt M.S., Callaway C.W., Carson A.P., Chamberlain A.M., Cheng S., Delling F.N. (2021). Heart Disease and Stroke Statistics—2021 Update: A Report from the American Heart Association. Circulation.

[10] Franquesa M., Pujol-Busquets G., García-Fernández E., Rico L., Shamirian-Pulido L., Aguilar-Martínez A., Medina F.X., Serra-Majem L., Bach-Faig A. (2019). Mediterranean Diet and Cardiodiabesity: A Systematic Review through Evidence-Based Answers to Key Clinical Questions. Nutrients.

[11] Martinez-Gonzalez M.A., Bes-Rastrollo M., Serra-Majem L., Lairon D., Estruch R., Trichopoulou A. (2009). Mediterranean Food Pattern and the Primary Prevention of Chronic Disease: Recent Developments. Nutr Rev..

[12] Martínez-González M.A., Gea A., Ruiz-Canela M. (2019). The Mediterranean Diet and Cardiovascular Health: A Critical Review. Circ. Res..

[13] Frieden T.R. (2017). Evidence for Health Decision Making—Beyond Randomized, Controlled Trials. N. Engl. J. Med..

[14] Multiple Risk Factor Intervention Trial Research Group (1982). Multiple Risk Factor Intervention Trial: Risk Factor Changes and Mortality Results. JAMA.

[15] McKinney M., Bell R., Samborski C., Attwood K., Dean G., Eakle K., Yu W., Edge S.B. (2021). Clinical Trial Participation A Pilot Study of Patient-Identified Barriers. Clin. J. Oncol. Nurs..

[16] Price A., Albarqouni L., Kirkpatrick J., Clarke M., Liew S.M., Roberts N., Burls A. (2018). Patient and Public Involvement in the Design of Clinical Trials: An Overview of Systematic Reviews. J. Eval. Clin. Pract..

[17] Thangaratinam S., Khan K.S. (2015). Participation in Research as a Means of Improving Quality of Care: The Role of a Principal Investigator in Multicentre Clinical Trials. Obstet. Gynaecol..

[18] Downer M.K., Gea A., Stampfer M., Sánchez-Tainta A., Corella D., Salas-Salvadó J., Ros E., Estruch R., Fitó M., Gómez-Gracia E. (2016). Predictors of Short- and Long-Term Adherence with a Mediterranean-Type Diet Intervention: The PREDIMED Randomized Trial. Int. J. Behav. Nutr. Phys. Act..

[19] Martínez-González M.A., Buil-Cosiales P., Corella D., Bulló M., Fitó M., Vioque J., Romaguera D., Alfredo Martínez J., Wärnberg J., López-Miranda J. (2019). Cohort Profile: Design and Methods of the PREDIMED-Plus Randomized Trial. Int. J. Epidemiol..

[20] O’Brien B.C., Harris I.B., Beckman T.J., Reed D.A., Cook D.A. (2014). Standards for Reporting Qualitative Research: A Synthesis of Recommendations. Acad. Med..

[21] Heidari S., Babor T.F., De Castro P., Tort S., Curno M. (2016). Sex and Gender Equity in Research: Rationale for the SAGER Guidelines and Recommended Use. Res. Integr. Peer Rev..

[22] Van Epps H., Astudillo O., Del Pozo Martín Y., Marsh J. (2022). The Sex and Gender Equity in Research (SAGER) Guidelines: Implementation and Checklist Development. Eur. Sci. Ed..

[23] Patton M.Q. (2014). Qualitative Research and Evaluation Methods: Integrating Theory and Practice.

[24] Haskell G.H., Headland T.N., Pike K.L., Harris M. (1992). Emics and Etics: The Insider/Outsider Debate. J. Am. Folk..

[25] Pope C., Mays N. (2020). Qualitative Research in Health Care.

[26] Phull S. (2015). The Mediterranean Diet: Socio-Cultural Relevance for Contemporary Health Promotion. Open Public Health J..

[27] Medina F.X., Solé-Sedeno J.M., Bach-Faig A., Aguilar-Martínez A. (2021). Obesity, Mediterranean Diet, and Public Health: A Vision of Obesity in the Mediterranean Context from a Sociocultural Perspective. Int. J. Environ. Res. Public Health.

[28] Counihan C., Pilcher J. (2012). Gendering Food. The Oxford Handbook of Food History.

[29] Fournier T., Jarty J., Lapeyre N., Touraille P. (2015). L’alimentation: Arme Du Genre. J. Anthropol..

[30] McPhail D., Beagan B., Chapman G.E. (2012). I Don’t Want to Be Sexist but "Denying and Re-Inscribing Gender through Food. Food Cult. Soc..

[31] Parsons J. (2015). Gender, Class, and Food: Families, Bodies and Health.

[32] Murcott A. (1986). The Sociology of Food and Eating: Essays on the Sociological Significance of Food.

[33] Murdock G.P., Provost C. (1973). Factors in the Division of Labor by Sex: A Cross-Cultural Analysis. Ethnology.

[34] Serra-Majem L., Tomaino L., Dernini S., Berry E.M., Lairon D., de la Cruz J.N., Bach-Faig A., Donini L.M., Medina F.X., Belahsen R. (2020). Updating the Mediterranean Diet Pyramid towards Sustainability: Focus on Environmental Concerns. Int. J. Environ. Res. Public Health.

[35] Braun V., Clarke V. (2022). Thematic Analysis: A Practical Guide.

[36] Hewitt-Taylor J. (2001). Use of Constant Comparative Analysis in Qualitative Research. Nurs. Stand..

[37] Annells M. (2006). Triangulation of Qualitative Approaches: Hermeneutical Phenomenology and Grounded Theory. J. Adv. Nurs..

[38] Dismore L., Hurst C., Sayer A.A., Stevenson E., Aspray T., Granic A. (2020). Study of the Older Adults’ Motivators and Barriers Engaging in a Nutrition and Resistance Exercise Intervention for Sarcopenia: An Embedded Qualitative Project in the MIlkMAN Pilot Study. Gerontol. Geriatr. Med..

[39] Rose L., Wood A., Gill T. (2024). Gender Differences in Adherence and Retention in Mediterranean Diet Interventions with a Weight-Loss Outcome: A Systematic Review and Meta-Analysis. Obes. Rev..

[40] Raparelli V., Romiti G.F., Spugnardi V., Borgi M., Cangemi R., Basili S., Proietti M., Lenzi A., Tiberti C., Panimolle F. (2020). Gender-Related Determinants of Adherence to the Mediterranean Diet in Adults with Ischemic Heart Disease. Nutrients.

[41] Basora J., Villalobos F., Pallejà-Millán M., Babio N., Goday A., Castañer O., Fitó M., Zomeño M.D., Pintó X., Sacanella E. (2020). Association between the Potential Influence of a Lifestyle Intervention in Older Individuals with Excess Weight and Metabolic Syndrome on Untreated Household Cohabitants and Their Family Support: The Predimed-plus Study. Nutrients.

[42] Marshall N., Butler M., Lambert V., Timon C.M., Joyce D., Warters A. (2025). Health Literacy Interventions and Health Literacy-Related Outcomes for Older Adults: A Systematic Review. BMC Health Serv. Res..

[43] Osborne R.H., Elmer S., Hawkins M., Cheng C.C., Batterham R.W., Dias S., Good S., Monteiro M.G., Mikkelsen B., Nadarajah R.G. (2022). Health Literacy Development Is Central to the Prevention and Control of Non-Communicable Diseases. BMJ Glob. Health.

[44] Paukkonen L., Oikarinen A., Kähkönen O., Kyngäs H. (2022). Adherence to Self-Management in Patients with Multimorbidity and Associated Factors: A Cross-Sectional Study in Primary Health Care. J. Clin. Nurs..

[45] Krukowski R.A., West D.S., Priest J., Ashikaga T., Naud S., Harvey J.R. (2019). The Impact of the Interventionist–Participant Relationship on Treatment Adherence and Weight Loss. Transl. Behav. Med..

[46] Hawke L.J., Nelson E., O’Brien P., Crossley K.M., Choong P.F., Bunzli S., Dowsey M.M. (2024). Influences on Clinical Trial Participation: Enhancing Recruitment through a Gender Lens—A Scoping Review. Contemp. Clin. Trials Commun..

[47] Yong C., Suvarna A., Harrington R., Gummidipundi S., Krumholz H.M., Mehran R., Heidenreich P. (2023). Temporal Trends in Gender of Principal Investigators and Patients in Cardiovascular Clinical Trials. J. Am. Coll. Cardiol..

[48] Denby K.J., Szpakowski N., Silver J., Walsh M.N., Nissen S., Cho L. (2020). Representation of Women in Cardiovascular Clinical Trial Leadership. JAMA Intern. Med..

[49] Apostolaki I., Pepa A., Vlassopoulos A., Kapsokefalou M. (2021). Social Capital and Self-Perceived Quality of Life-Interrelated Predictors of Mediterranean Diet Adherence in Older Adults. Nutrients.

[50] Surrow S., Jessen-Winge C., Ilvig P.M., Christensen J.R. (2021). The Motivation and Opportunities for Weight Loss Related to the Everyday Life of People with Obesity: A Qualitative Analysis within the DO:IT Study. Scand. J. Occup. Ther..

[51] Murray J., Fenton G., Honey S., Bara A.C., Hill K.M., House A. (2013). A Qualitative Synthesis of Factors Influencing Maintenance of Lifestyle Behaviour Change in Individuals with High Cardiovascular Risk. BMC Cardiovasc. Disord..

[52] Trujillo-Garrido N., Santi-Cano M.J. (2022). Motivation and Limiting Factors for Adherence to Weight Loss Interventions among Patients with Obesity in Primary Care. Nutrients.

[53] Baer N.R., Zoellick J.C., Deutschbein J., Anton V., Bergmann M.M., Schenk L. (2021). Dietary Preferences in the Context of Intra-Couple Dynamics: Relationship Types within the German NutriAct Family Cohort. Appetite.

[54] Cobb L.K., Godino J.G., Selvin E., Kucharska-Newton A., Coresh J., Koton S. (2016). Spousal Influence on Physical Activity in Middle-Aged and Older Adults. Am. J. Epidemiol..

[55] Albanese A.M., Huffman J.C., Celano C.M., Malloy L.M., Wexler D.J., Freedman M.E., Millstein R.A. (2019). The Role of Spousal Support for Dietary Adherence among Type 2 Diabetes Patients: A Narrative Review. Soc. Work. Health Care.

[56] Navarro-Martínez R., Mafla-España M.A., Cauli O. (2022). Mediterranean Diet Adherence in Community-Dwelling Older Adults in Spain: Social Determinants Related to the Family. Nutrients.

