# Supplementary material for: Participants’ Perspectives on Health Impact, Barriers and Facilitators to Adherence in a Mediterranean Diet Lifestyle Trial
Source: Nutrients. 2025 Dec 24;18(1):63. doi: 10.3390/nu18010063 (PMC12787583; doi:10.3390/nu18010063)
Supplement: Supplementary file 1 [file nutrients-18-00063-s001.zip › Supplement S1.pdf]

ease publications

Sex and Gender Equity in Research Guidelines Checklist

EASE Gender Policy Committee

The Sex and Gender Equity in Research (SAGER) Guidelines[1] are a comprehensive procedure for reporting of sex and gender information in study design, data analysis, results and interpretations of findings. They are primarily designed to guide authors in preparing their manuscripts but they are also useful for editors to integrate assessment of sex and gender in all manuscripts as an integral part of the editorial process.

The two checklists in this document are developed from the guidelines presented in the original SAGER article, with additional requirements identified by some *Lancet* journals.[2] They present a convenient list of items to check off when writing, reviewing or editing manuscripts.

One checklist is intended for studies including human participants and one for studies that do not include human participants, such as those using animals and cells.

This checklist was originally published as an article in the EASE journal, European Science Editing[2].

References

1. Heidari, S., Babor, T.F., De Castro, P., Tort S., Curno M. (2016) Sex and Gender Equity in Research: Rationale for the SAGER Guidelines and Recommended Use. Research Integrity and Peer Review, **1:** 2. https://doi.org/10.1186/s41073- 016-0007-6

2. Van Epps H, Astudillo O, Del Pozo Martin Y, Marsh J (2022) The Sex and Gender Equity in Research (SAGER) Guidelines: Implementation and Checklist Development. European Science Editing, 48: E86910. https://doi.org/10.3897/ese.2022.e86910

Authors

This checklist was created by Heather Van Epps*, Olaya Astudillo*, Yaiza Del Pozo Martín, and Joan Marsh, of the EASE Gender Policy Committee.

*Joint first authorship

Affiliations:
The Lancet Rheumatology (H Van Epps)
The Lancet (O Astudillo)
The Lancet Haematology (Y Del Pozo Martín)
The Lancet Psychiatry (J Marsh)

About the EASE Gender Policy Committee

Established in 2012, the EASE Gender Policy Committee works to advance gender- and sex-sensitive reporting and communication in science. The goal is not only better science, whether in the life, natural or social sciences, but also enhanced evidence-based practices, interventions and opportunities, for everyone. The Committee consists of experts with diverse backgrounds, linguistic traditions and professional experience, sharing enthusiasm to advance sex and gender equity for responsible research and innovation.

Read more about the committee on their EASE Community web pages: https://ease.org.uk/communities/gender-policy-committee/about-this-committee/

Table 1. SAGER guidelines checklist
Studies with human participants

Section / topic	Item number	Checklist item	Reported on page number
**General**			
	1	The terms sex/gender used appropriately	1-14
**Title**			
	2	Title specifies the sex/gender of participants if only one included	Not Applicable
**Abstract**			
	3a	Abstract specifies the sex/gender of participants if only one included	Not Applicable
	3b	Study population described with sex/gender breakdown*	4-6 (Results)
**Introduction**			
	4a	If relevant, previous studies that show presence or lack of sex/gender differences or similarities are cited	2
	4b	Mention of whether sex/gender might be an important variant and if differences might be expected	2
	4c	The demographics of the study population with regard to sex/gender (eg, disease prevalence among male/female study participants) are outlined*	4-6 (Results)
**Methods**			
	5a	Method of definition of sex/gender (eg, self-report, genetic testing)	Not Reported
	5b	Description of how sex/gender was considered in the design, whether authors ensured adequate representation of male and female study participants, justification of the reasons for any exclusion of male or female participants, or explanation if not considered. Justification of other sex/gender-specific interventions of study designs (eg, mandating contraception for women).* Explicit reporting of the scientific rationale for	3-4

		contraception requirements and exclusions for pregnancy and lactation should be required*	
**Results**			
	6a	Study population description with complete gender/sex breakdown for all categories considered*	4-6
	6b	Where appropriate, data presented disaggregated by sex/gender, and sex/gender differences and similarities are described	6-13
	6c	Sex- and gender-based analyses reported regardless of outcome (in main paper if pre-specified; otherwise in appendix)*	6-13
	6d	For clinical trials, adverse event data disaggregated by sex/gender (in main paper if pre-specified; otherwise in appendix)*	Not Applicable
	6e	Patient-reported outcome data disaggregated by sex/gender (in main paper if pre-specified; otherwise in appendix)*	6-13
	6f	For epidemiological studies, the effects of other exposures on health problems examined for all genders and analysed critically from a gender perspective	Not Applicable
	6g	Table 1 includes separate rows for male sex/gender, female sex/gender and other categories if collected*	Not Applicable
**Discussion**			
	7a	Potential implications of sex/gender on the study results and analyses, including the extent to which the findings can be generalized to all sexes/genders in a population	13-14
	7b	If a sex/gender analysis not done, a rationale is given and implications of the lack of such analysis on the interpretation of the results are discussed	Not Applicable

Adapted from SAGER guidelines. Sex and Gender Equity in Research: rationale for the SAGER guidelines and recommended use. Research Integrity and Peer Review 1, Article number: 2 (2016) https://researchintegrityjournal.biomedcentral.com/articles/10.1186/s41073-016-0007-6.
* These points extend beyond the original SAGER table
