# Supplementary material for: Participants’ Perspectives on Health Impact, Barriers and Facilitators to Adherence in a Mediterranean Diet Lifestyle Trial
Source: Nutrients. 2025 Dec 24;18(1):63. doi: 10.3390/nu18010063 (PMC12787583; doi:10.3390/nu18010063)
Supplement: Supplementary file 1 [file nutrients-18-00063-s001.zip › Supplement S2.pdf]

Supplement S2. Topic guide of semi-structured interviews to participants in the qualitative study.

Topics	Questions
Sociodemographic characteristics	Where do you live?<br><br>Are you married?<br><br>Do you live alone or with housemates?<br><br>What is your profession? Are you retired?
Experiences on participation in the MedDiet clinical trial	What expectations did you have when you enrolled in the clinical trial? Do you think expectations have been met?<br><br>When you started the study program, did you set any personal goals or objectives?<br><br>Are you satisfied with the achievements or changes made through your participation in the trial?<br><br>What aspects of the clinical trial would you highlight?<br><br>What aspects did you not like?<br><br>Did you have any difficulties attending the meetings?<br><br>Did you find it easy to complete the questionnaires or undergo the tests?<br><br>What did you miss or find lacking?<br><br>What would you change about the study protocol?<br><br>Would you have liked to participate in its design?<br><br>When the clinical trial ends, do you think you will manage to maintain your new habits on your own?
Health perception	How would you assess your overall health — physically, mentally, and socially?<br><br>Is there any aspect of your health that concerns you?<br><br>Are you satisfied with your current weight and physical appearance?
Adherence to diet recommendations	Do you think you have gained new knowledge about healthy eating?<br><br>Did you hold any mistaken beliefs before enrolling in the clinical trial? Did you think that your diet was healthy?<br><br>Do you like to stay informed about nutrition-related topics? Do you usually exchange recipes with friends?

	Do you enjoy food? Do you like cooking?<br><br>Do you often share meals with your family?<br><br>Do you think your food tastes or preferences have changed?<br><br>Do you think you eat better now? Have you changed your way of eating? Is it easy for you to follow dietary recommendations in your daily meals?<br><br>Have you encountered any difficulties in following the recommendations due to health or other issues?<br><br>Do you think your household members have also changed their habits?
Adherence to physical activity recommendations	Do you engage in physical activity regularly?<br><br>Do you exercise with someone else?<br><br>Do you have any difficulty engaging in physical activity?
