# Supplementary material for: Participants’ Perspectives on Health Impact, Barriers and Facilitators to Adherence in a Mediterranean Diet Lifestyle Trial
Source: Nutrients. 2025 Dec 24;18(1):63. doi: 10.3390/nu18010063 (PMC12787583; doi:10.3390/nu18010063)
Supplement: Supplementary file 1 [file nutrients-18-00063-s001.zip › Table S1 .pdf]

Table S1. Theme: Experiences of enrollment in MedDiet lifestyle trial

Category	Subcategory	Illustrative quotes
**1. Motivations for participation**	**Health and diet literacy**	"Being taught to eat well, because we don't know how to eat well." (08399, Woman, 60 years) "I am interested in … the correlation between certain diseases, diet, and weight." (08241, Man, 60 years)
	**Self-control and self-acceptance**	"…improving one's conscience in terms of diet, exercise and leading a healthy life." (08382, Man, 61 years) "And it was very good for me to come here… I started coming here and thinking, and I lost 14 kg." (08027, Woman, 67 years)
	**Commitment to society and research**	"…if this is a valuable study, first for some people who might fry their eggs with butter, so that they learn not to do so." (08341, Woman, 63 years) "The first point is collaboration with young people, ... so that girls can get scholarships and … have a future." (08341, Woman, 63 years)
	**Counselling for age-related problems**	"We need help ... to cope with ... all the problems that arise at our age and we … don't know how to solve them." (08181, Woman, 70 years)
	**Cultural pride and Mediterranean identity**	"…if we're going to promote olive oil and the Mediterranean diet, then let's export the way of life as well." (08382, Man, 61 years)
	**Passive expectancy and initial uncertainty**	"I had no idea what it was. I was like a person starting something new, like gymnastics when you go blind on the first day." (08093, Woman, 67 years)
	**Perceived burden of study duration**	"They gave me some information papers… I read them and said: 'Wow, four years! Well, we'll see how this turns out'. But over time … I found it all great." (08181, Woman, 70 years)
**2. Challenges and personal goals**	**Lack of personal goals and reliance on researchers**	"The goal was the one they set for you: to lose weight… following the guidelines they set." (08347, Man, 69 years)
	**Self-confidence and perseverance**	"Personally, to carry it out… They asked me: 'What weight would you like to reach?' And I said, '60-62 kg'. Well, I'm working on it." (08200, Woman, 67 years) "My option is to maintain my weight, which has been fabulous with this program." (08382, Man, 61 years)
	**Desire for autonomy and self-sufficiency**	"I just want to be healthy and be able to walk and take care of myself … I don't want to burden my daughters." (08381, Woman, 76 years)
	**Frustration and loss of motivation**	"I saw this as a dream … because I was about twenty-something kilos overweight." (08200, Woman, 67 years) "At first, I set myself some goals, but then I didn't set myself anymore." (08212, Man, 70 years)
