# Supplementary material for: Participants’ Perspectives on Health Impact, Barriers and Facilitators to Adherence in a Mediterranean Diet Lifestyle Trial
Source: Nutrients. 2025 Dec 24;18(1):63. doi: 10.3390/nu18010063 (PMC12787583; doi:10.3390/nu18010063)
Supplement: Supplementary file 1 [file nutrients-18-00063-s001.zip › Table S2.pdf]

Table S2. Theme: Participants' opinions and suggestions for improving the intervention

Category	Subcategory	Participants' verbatim quotes
**5.1. Overall satisfaction and trust in the research design**	**No need for change**	"I've been fine. I don't see any negative aspects or things that you might say we could improve. I haven't thought about that issue (...) Besides, as you are all very pleasant in general, we always adapt [to the study protocol tests]. I have always tried to cause as little disruption as possible and attend my meetings to contribute. I have no complaints or objections whatsoever." (08212, Man, 70 years) "The truth is that (...) I can't find any faults [with the study]. For me, everything I've seen is very, very, very positive. I (...) think (...) that it's a real gift, how it's been planned, how those in charge of it are running it, the details of the extra oil. I don't know, everything is very good, I think everything has been very, very well thought out, very well studied and with experience." (08200, Woman, 67 years) "I think the study is good (...) it is well run, well planned, we adapt to what there is." (08351, Man, 74 years)
	**Acriticism, conformity, and passive role**	"It's them [the researchers] who have to say. Not me, I don't feel qualified to do that. I thought she was very proper, very polite, very approachable." (08341, Woman, 63 years) "No, I don't consider myself competent for that." (08241, Man, 60 years) "Personally, I wouldn't change anything, nor have I seen anything (...) I can't complain about anything, nor would I correct anything. Maybe there are things among you that could be improved, but for me, no, I'm quite satisfied." (08347, Man, 69 years) "No. I have no authority whatsoever. I found everything to be very coherent. (...) I think it's very good. You know why things are planned and why certain things are done and so on." (08200, Woman, 67 years)
	**Own responsibility for weight gain**	"I don't know what could be improved because what you've told me and done has been perfect, it's worked for me. (...) If I've ever messed up a little bit, well, it hasn't worked for me because there's no going back. And at the same time, I don't want that because it hurts me." (08032, Man, 81 years)

**5.2. Unmet expectations and perceived gaps in the intervention**	**Psychological care focused on older adults, rather than emphasis on nutrition**	"I would have appreciated it if they had given us a (...) I thought we were going to get some help with (...) age-related problems, because they do exist, living together (...) each person's behaviour (...) how we treat each other (...), some information (...), a psychologist or someone we could ask questions about that... but we didn't get it. (...) this has focused a lot on food, hasn't it? More than anything else, I noticed a lack because, well, this has dealt with many things, but the focus has been on food. I noticed, well, it would have been nice (...) if we had had a little chat with someone. (...) The thing is that it [the study] focused more on food, so in that respect, I've never had a problem, because our diet has always been more or less Mediterranean, as we say (...) This has only been about nutrition, and it was clear from the beginning that it was going to be about nutrition, but I don't know, I was thinking, I mean. Over the years, as it's going to be many years, over time... That's something I've missed." (08181, Woman, 70 years)
	**Plain language in group sessions**	"I find it very technical, which may not be accessible to many people. For example, my brother Juan is a chemist and he's a genius, so he knows everything you're talking about in general, lots of things that most people don't know about. Why? Because we're not prepared to be part of the dynamic you're talking about." (08212, Man, 70 years)
